# Usefulness of Low-Resistance Electrodes Used in Iontophoresis to Enhance Skin Permeability of Drugs

**DOI:** 10.3390/molecules31183269

**Published:** 2026-09-15

**Authors:** Takeshi Oshizaka, Yuya Yanagibashi, Yuki Matsui, Yusaku Kachi, Issei Takeuchi, Kenji Mori, Kenji Sugibayashi

**Affiliations:** 1 Faculty of Pharmaceutical Sciences, Josai International University, 1 Gumyo, Togane 283-8555, Chiba, Japan; oshizaka@jiu.ac.jp (T.O.); itakeuchi@jiu.ac.jp (I.T.); 2 Faculty of Pharmacy and Pharmaceutical Sciences, Josai University, 1–1 Keyakidai, Sakado 350-0295, Saitama, Japan

**Keywords:** skin permeation, iontophoresis, low-resistance electrodes, electric current, lidocaine

## Abstract

This study investigated how electrode design and electrical conditions influence iontophoretic transdermal drug delivery. A low-resistance silver electrode (Ag*) was developed to reduce interfacial resistance and improve the delivery of lidocaine hydrochloride (LID·HCl). In vitro permeation experiments were conducted using pig ear skin mounted on diffusion cells. Iontophoresis was applied under constant current, constant voltage, and battery-powered conditions. Ag* and conventional Ag electrodes were compared as anodes, with an Ag/AgCl cathode. Drug permeation and current profiles were measured over 8 h. Under constant current conditions, LID·HCl permeation through skin increased linearly with current density (r = 0.993). Enhanced permeation was also observed under constant voltage and battery-driven systems. The Ag* electrode produced higher current and greater drug permeation than the conventional Ag electrode at the same voltage due to its microstructured surface, which increased effective surface area and reduced charge-transfer resistance. Skin permeation was governed by current intensity. The Ag* electrode enabled efficient drug delivery even with simple battery systems, suggesting its potential for use in wearable transdermal delivery devices.

## 1. Introduction

Corneocytes in the stratum corneum, the outermost layer of the skin, are terminally differentiated cells derived from keratinocytes in the viable epidermis. During their upward migration, keratinocytes lose their nuclei and intracellular organelles and become non-viable cells composed primarily of the rigid structural protein keratin. The stratum corneum exhibits a called brick and mortar structure, in which approximately 10–20 layers of corneocytes are stacked [1,2]. Intercellular spaces are densely filled with lipids, mainly ceramides, cholesterol, and free fatty acids, which are organized into lamellar structures [3,4]. Consequently, the stratum corneum functions as an effective barrier, preventing transepidermal water loss as well as the penetration of external substances such as bacteria and viruses [4,5,6]. On the other hand, the skin is widely used as a site for the application of pharmaceuticals and cosmetics. Chemical substances applied to the skin can penetrate or permeate it and may exert pharmacological effects either locally or systemically [7]. To achieve a pharmacological effect via dermal application, the concentration of the chemical substance at the site of action is crucial [8,9,10]. However, because the stratum corneum possesses a strong barrier function, only a limited number of chemical substances can penetrate or permeate the skin [11].

Therefore, in order to enhance the amount and rate of skin penetration and permeation of active ingredients in pharmaceuticals and medicinal cosmetics, various chemical and physical penetration enhancement methods have been investigated. Chemical penetration enhancement methods include *l*-menthol [12], ethanol [13], fatty acids [14], surfactants [15], urea [16], and Azone [17]. We have recently reported a chemical enhancement approach using an ante-enhancer with a structure similar to Azone [18,19]. On the other hand, physical penetration enhancement methods include iontophoresis [20,21], electroporation [22], microneedles [23], sonophoresis [24], and plasma irradiation [25,26]. In recent years, combination approaches such as iontophoresis or electroporation with microneedles have been reported [27], as well as remote-controlled iontophoresis systems have also been reported, in which the drug dosage can be adjusted even when healthcare professionals are not in close proximity to the user [28,29].

Iontophoresis is a method in which a weak electric current is applied to the skin to enhance the transdermal delivery of ionized chemical substances via electrorepulsion [30]. During iontophoresis, ions, including ionized drug molecules and endogenous ions present in the skin, migrate under the influence of the applied electric field through electrorepulsion. In addition, electroosmosis is induced, whereby water moves within the skin together with the transport of both ionized and non-ionized compounds [31]. However, the electroosmosis effect is generally considered to be weaker than the electrorepulsion effect [32,33]. Iontophoresis has been applied to various chemical substances in practical use: fentanyl, lidocaine, and sumatriptan in the pharmaceutical field [34,35,36,37,38] and vitamin C and related compounds in the cosmetic field [39,40,41]. In previous fundamental studies on iontophoresis, large-scale devices were commonly employed [41]. However, recent developments have focused on compact systems incorporating silver electrodes embedded in beauty masks [41,42].

**Figure 1:**
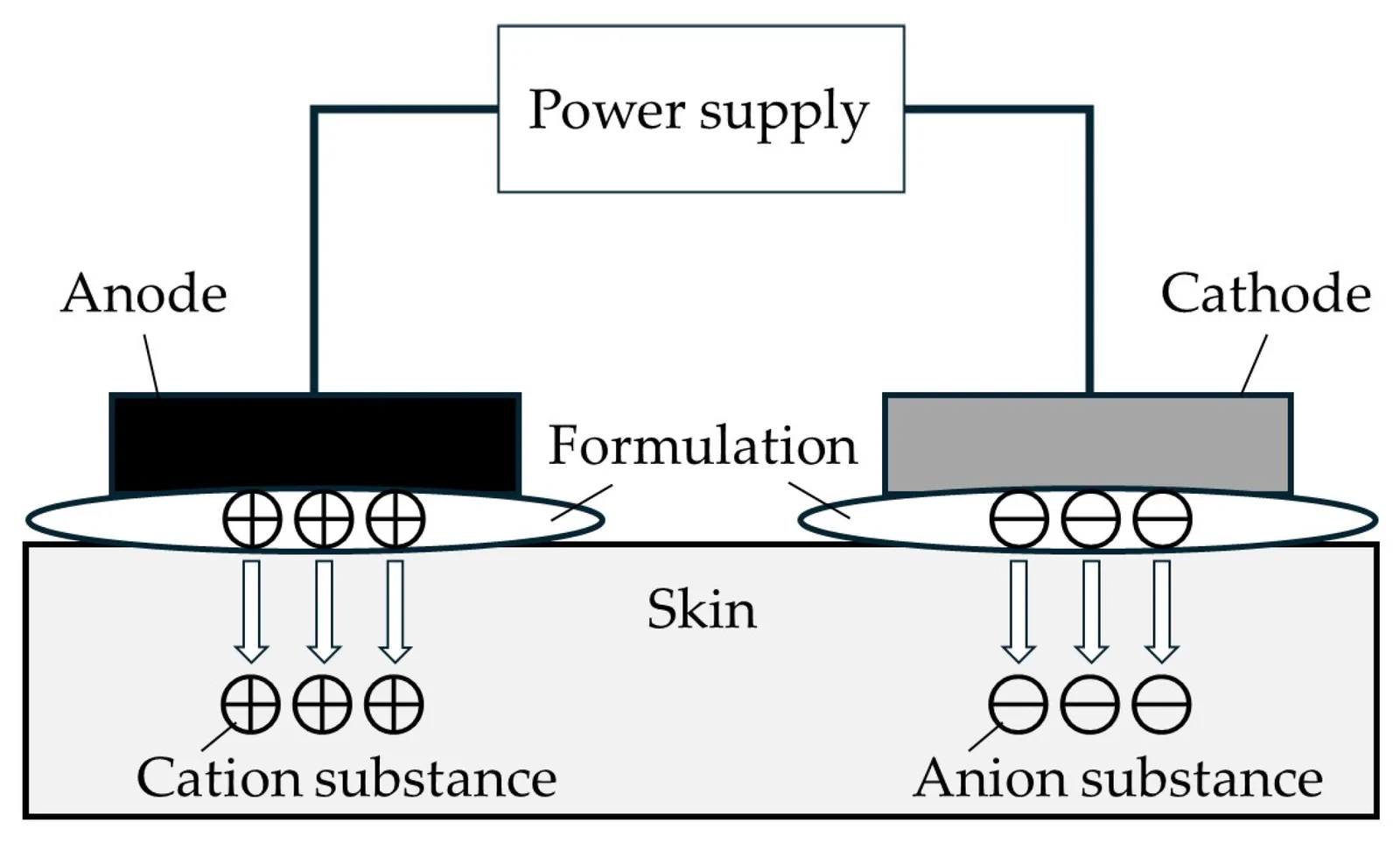
Schematic diagram illustrating the principle of iontophoresis.

Generally, in iontophoresis, an electrode pad containing a drug and a reference electrode pad are placed on the skin, and an electric current is applied using a power supply to enhance transdermal drug delivery via electrorepulsion (Figure 1). However, electrical resistance can arise at the electrode–chemical compound layer interface, the electrode–electrolyte layer interface, the chemical compound layer–skin interface, or the electrolyte layer–skin interface, which may hinder current flow and consequently reduce chemical compound permeation. Therefore, we hypothesized that reducing the overall resistance of the system would facilitate the current flow and drug permeation. In this study, we first focused on developing a low-resistance electrode to reduce electrical resistance at the electrode–chemical compound layer interface. Furthermore, we considered that lowering electrode resistance would enable iontophoretic delivery even with a low driving force such as dry batteries and thus aimed to simplify the system using D and AA batteries. Lidocaine hydrochloride (LID·HCl) was used as a model compound.

## 2. Materials and Methods

### 2.1. Materials

LID·HCl was purchased from Sigma-Aldrich (St. Louis, MO, USA). Sodium chloride, phosphoric acid, acetonitrile, and sodium *n*-dodecyl sulfate were obtained from Kanto Chemical Co., Inc. (Tokyo, Japan). A direct current generator (VI1002) was purchased from Precise Gauges Co., Ltd. (Shizuoka, Japan). Dry batteries (D battery: LR20N/4S and AA battery: LR6EXD/4S, each with an electromotive force of 1.5 V) were obtained from Mitsubishi Electric Corporation (Tokyo, Japan). Silver foil used as the electrode for iontophoresis was purchased from Murata Yohaku & Co. (Tokyo, Japan). All other reagents and solvents were used without further purification.

### 2.2. Electrode Preparation

**Figure 2:**
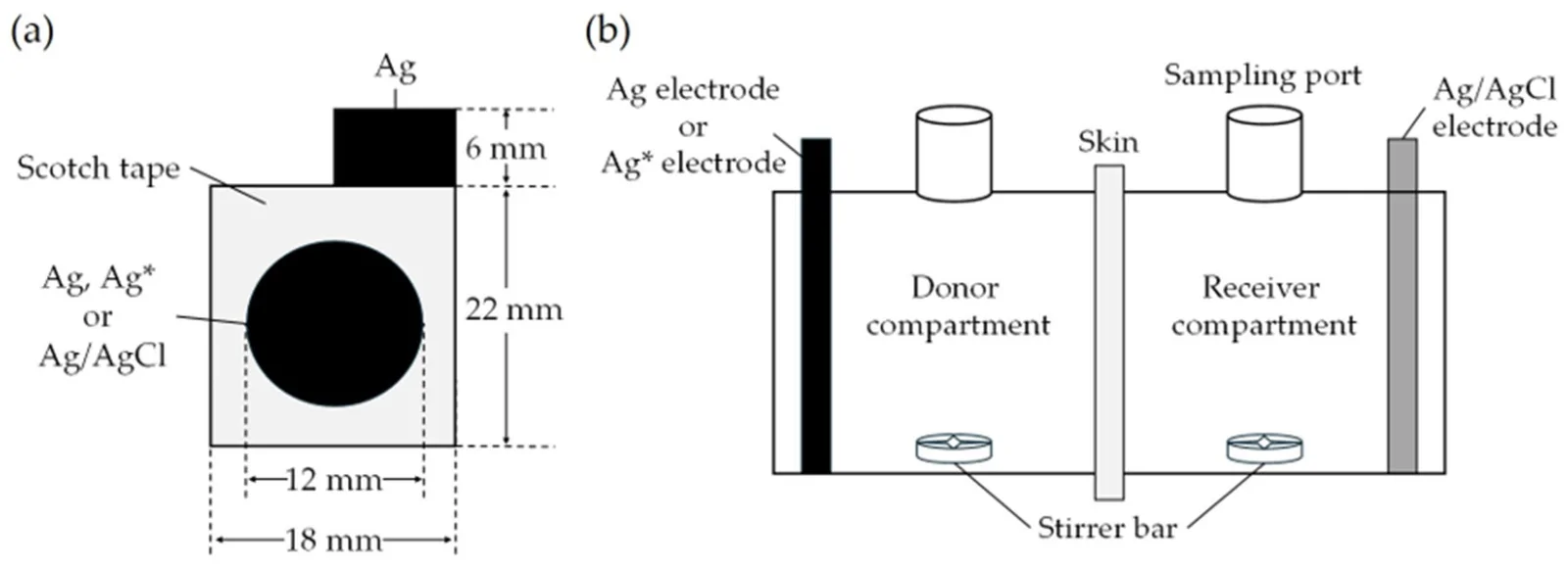
Schematic diagram of electrodes (**a**) and horizontal two-chamber diffusion cells used in this experiment (**b**).

Figure 2 shows the electrodes and the horizontal two-chamber diffusion cell used in this study. A silver foil was cut into a rectangular shape (18 × 22 mm) with a protruding section (9 × 6 mm) (Figure 2a). The cut silver foil was then covered with cellophane tape (Nichiban Co., Ltd., Tokyo, Japan) with a circular opening of 12 mm in diameter at the center, yielding the Ag electrode. The Ag electrode was used as the anode (Figure 2b).

**Figure 3:**
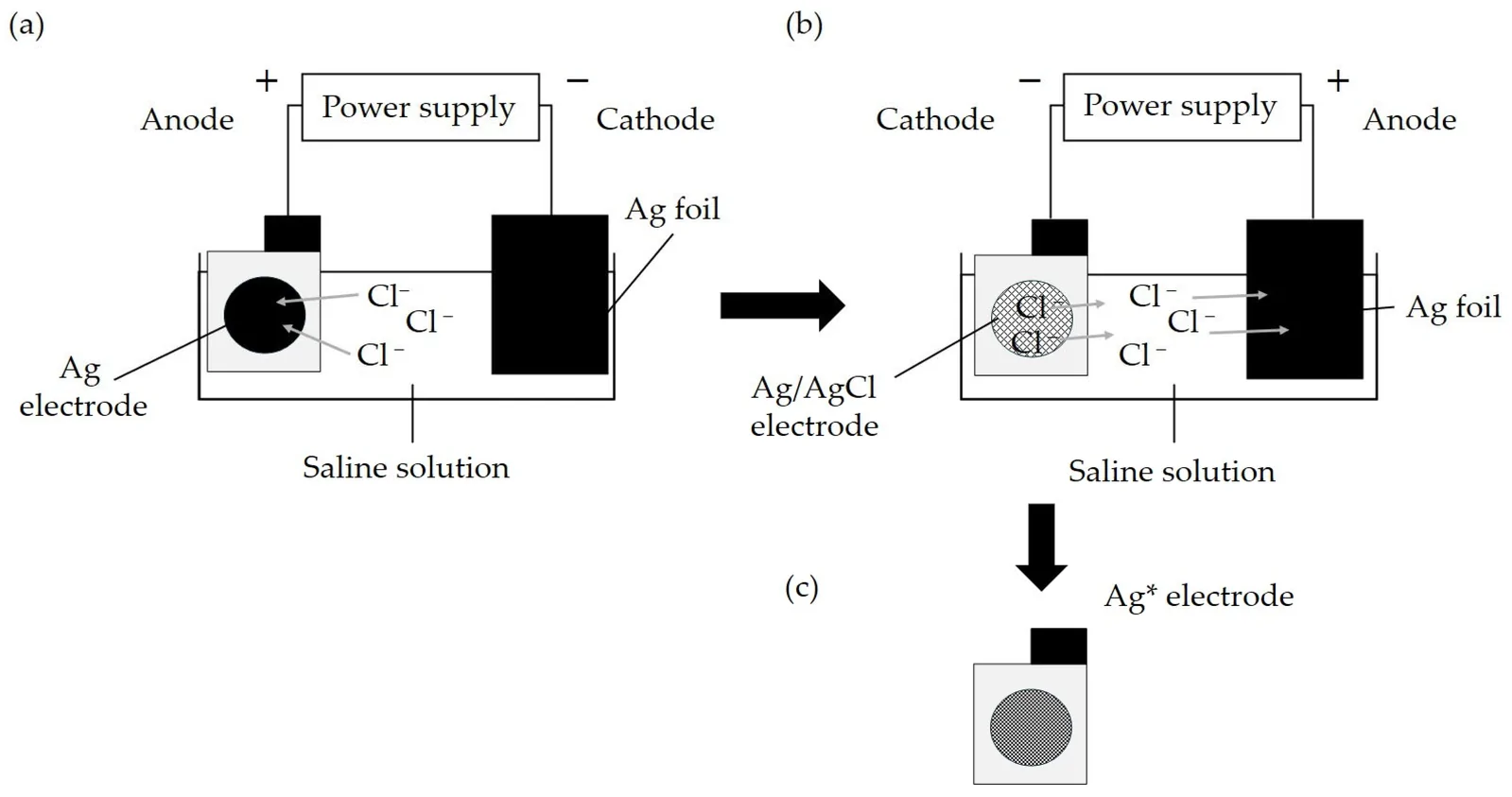
The electrode preparation process of the Ag/AgCl electrode (**a**) and the Ag electrode (**b**). Ag* electrode (**c**).

Figure 3 shows the electrode preparation process of the Ag/AgCl electrode and the Ag electrode. The Ag/AgCl electrode was prepared by connecting silver foil to the cathode side and the Ag electrode to the anode side of a direct current generator (Figure 3a). The circular portions of both the silver foil and the Ag electrode were immersed in saline, and a current density of 0.2 mA/cm^2^ was applied for 16 h.

The low-resistance electrode (Ag* electrode) was prepared by placing the Ag/AgCl electrode and silver foil on the cathode and anode sides of the direct current generator, respectively (Figure 3b). In addition, the Ag* electrode was obtained by immersing the circular portions of both the silver foil and the Ag/AgCl electrode in saline and applying a current density of 0.2 mA/cm^2^ for 16 h (Figure 3c). Based on the total charge applied during iontophoresis, the charge used for AgCl and Ag* formation was set at 3.2 mA·h/cm^2^.

### 2.3. Scanning Electron Microscope (SEM) Observation of the Ag and Ag* Electrode

The surface morphology of the Ag electrode and the prepared Ag* electrode was observed using a scanning electron microscope (S-3000N; JEOL, Tokyo, Japan) without gold coating.

### 2.4. Measurement of the Charge Transfer Resistance at Ag and Ag* Interfaces Using Electrochemical Impedance Spectroscopy (EIS)

**Figure 4:**
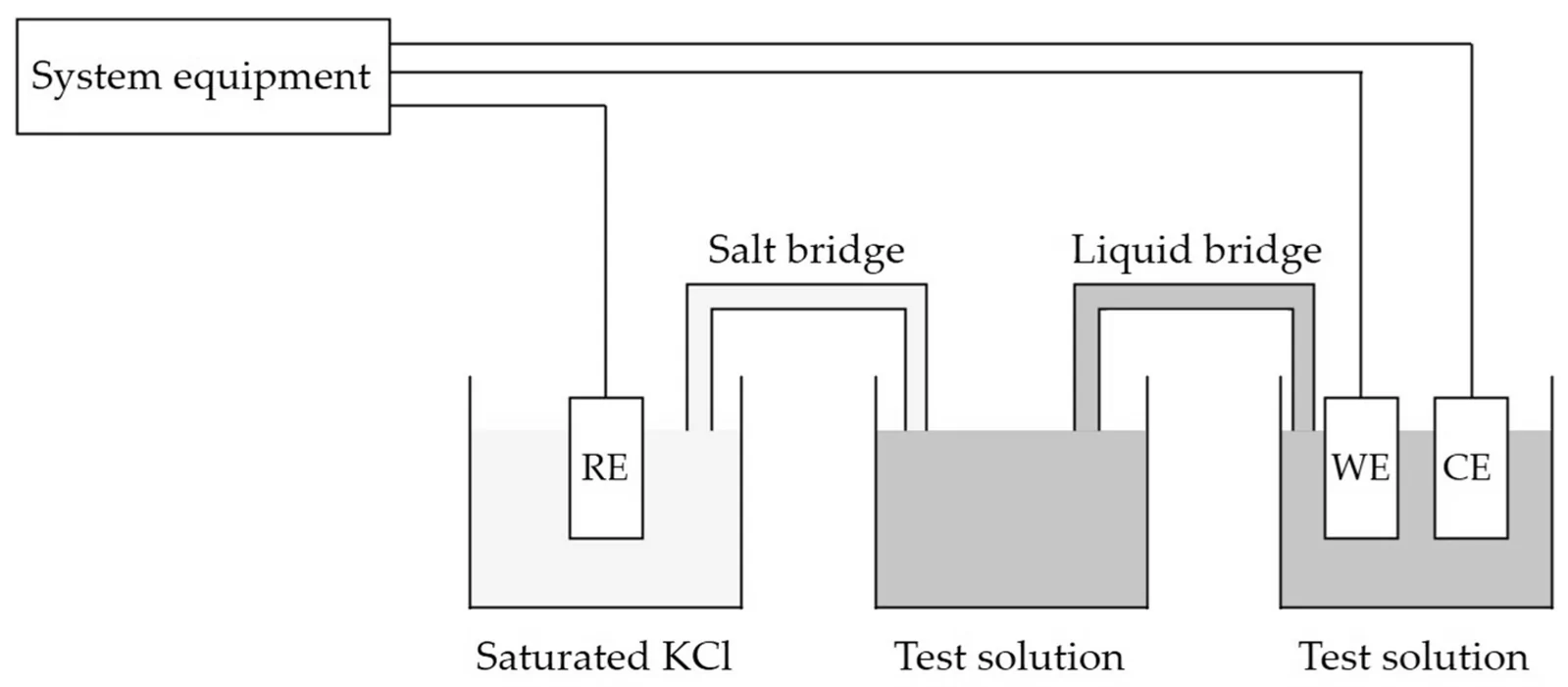
A schematic diagram of the EIS measurement system. Abbreviations: WE; working electrode, CE; counter electrode, and RE; reference electrode.

Electrochemical impedance spectroscopy (EIS) was performed using Ag and Ag* electrodes as the working electrode, and the solution resistance and interfacial charge-transfer resistance were determined from the Nyquist plots (see below). A schematic diagram of the EIS measurement system is shown in Figure 4.

A multichannel electrochemical measurement system (HZ-Pro S2A, Meidensha Hokuto Co., Ltd., Tokyo, Japan) was used for the EIS measurements. A platinum electrode and an Ag/AgCl electrode were used as the counter electrode and reference electrode, respectively.

The test solution consisted of an aqueous solution containing 5 mM potassium ferricyanide, 5 mM potassium ferrocyanide, and 0.1 M potassium chloride. Measurements were conducted at 37 °C with an AC voltage amplitude of 10 mV over a frequency range from 100 kHz to 10 mHz.

The measurements in this study were conducted by JAPAN TESTING LABORATORIES, Inc. (Gifu, Japan).

### 2.5. Analysis Methods of Nyquist Plot Data [43]

The experimentally obtained Nyquist plot data were fitted using a nonlinear least-squares method based on the Levenberg–Marquardt algorithm.

For the Ag* electrode, the data were fitted using Equation (1) based on a two-time-constant equivalent circuit, *R_s_* − (*R*_1_||*C*_1_) − (*R*_2_||*C*_2_) and the values of *R*_1_ and *R*_2_ were determined. In contrast, for the Ag electrode, the two-time-constant equivalent circuit alone was insufficient to adequately describe the impedance behavior in the low-frequency region. Therefore, because the contribution of a diffusion process was observed in the low-frequency region of the Nyquist plot, an equivalent circuit including a Warburg element (*W_d_*_1_), *R_s_* − (*R*_1_||*C*_1_) − ((*R*_2_+*W_d_*_1_)||*C*_2_), was employed. The data were fitted using Equation (2), and the values of *R*_1_ and *R*_2_ were determined in the same manner.

(1) $$Z_{tot}=R_{s}+\frac{1}{j\omega C_{1}+\frac{1}{R_{1}}}+\frac{1}{j\omega C_{2}+\frac{1}{R_{2}}}$$

(2) $$Z_{tot}=R_{s}+\frac{1}{j\omega C_{1}+\frac{1}{R_{1}}}+\frac{1}{j\omega C_{2}+\frac{1}{R_{2}+R_{N1}\frac{tanh(\delta_{N}\sqrt{j\omega })}{\delta_{N}\sqrt{j\omega }}}}$$

where *Z_tot_* represents the total impedance of the system, *R_s_* represents the solution resistance, *j* is the imaginary unit, and *ω* is the angular frequency. *C*_1_ and *C*_2_ represent capacitance components. *R*_1_ represents the resistance component associated with a surface layer formed on the electrode surface, such as an oxide film or an adsorbed layer, whereas *R*_2_ represents the charge-transfer resistance at the electrode/electrolyte interface. In addition, *R_N_*_1_ is a resistance parameter associated with diffusion, and *δ_N_* is a parameter representing diffusion characteristics, such as the thickness of the diffusion layer. Furthermore, tanh denotes the hyperbolic tangent function. The Warburg element included in Equation (2) accounts for the mass-transfer (diffusion) process of redox species in the vicinity of the electrode surface. *R*_2_ was evaluated as a parameter corresponding to the charge-transfer resistance at the electrode/electrolyte interface.

### 2.6. Skin Permeation Experiment

Pig ear skin from male or female pigs (LWD, 5–6 months old) was obtained from the Feed and Livestock Central Research Laboratory, National Federation of Agricultural Cooperative Associations (Zen-Noh) (Tsukuba, Ibaraki, Japan). In this study, commercially obtained excised pig ears were used for experiments. Thus, the institutional ethics committee of the Josai International University judged that ethical review for animal experimentation was not required. Frozen pig ears were thawed at room temperature prior to use. After removing hair from the ears with scissors, the skin surface was washed with purified water. Excess subcutaneous fat attached to the dermis side was carefully removed, and the skin was used for permeation experiments.

The pig ear skin was mounted in a horizontal two-chamber diffusion cell (effective permeation area: 2.01 cm^2^, Figure 2b) with the stratum corneum facing the donor compartment (anode side). The donor and receptor compartments were hydrated with purified water for 1 h to equilibrate the skin. After hydration, the water was removed. The Ag or Ag* electrode, serving as the anode, was placed in the donor compartment, whereas the Ag/AgCl electrode, serving as the cathode, was placed in the receiver compartment. Subsequently, 10% LID·HCl aqueous solution was applied to the donor compartment, and approximately 5.0 mL of saline was added to the receiver compartment, after which the skin permeation study was initiated.

Table 1 summarizes the energizing devices, anode electrodes, and energizing conditions in the present iontophoresis experiments. In all cases, an Ag/AgCl electrode was used as the cathode. In the control group, no electrodes were applied, and skin permeation was evaluated using 10% LID·HCl solution applied to the donor compartment.

Cases 1–3 were performed under constant current conditions using a direct current generator with a Ag electrode placed on the anode side. Current densities of 0.0498, 0.124, and 0.199 mA/cm^2^ were applied, corresponding to cases 1, 2, and 3, respectively. Case 4 involved a constant voltage condition (1.5 V/effective diffusion area) using a Ag electrode on the anode side and a direct current generator. Cases 5 and 6 were conducted using a Ag electrode on the anode side and dry batteries with an electromotive force of 1.5 V (D battery and AA battery, respectively). Cases 7 and 8 were performed using the Ag* electrode on the anode side with D and AA batteries, respectively. In the battery-driven experiments, the applied voltage was equivalent to 1.5 V/effective diffusion area.

In all cases, current was applied continuously from the start of the permeation experiment until the end of the 8 h study period. During the experiments, the receptor solution was stirred using a magnetic stirrer to maintain sink conditions. At 1 h intervals, 1.0 mL samples were withdrawn from the receptor compartment and replaced with an equal volume of saline. The concentration of LID·HCl in the collected samples was determined by HPLC after deproteinization.

### 2.7. Electric Current Measurement

In cases 4–8, at each time point (*i*) after the start of the skin permeation experiment, a 100 Ω resistor was connected in series to the iontophoresis circuit, and the voltage (*V_i_*) was measured using an oscilloscope (Scope Corder DL750, Yokogawa Test & Measurement Co., Tokyo, Japan). The current (*I_i_*) was then calculated from *V_i_* using Equation (3).

(3) $$I_{i}=\frac{V_{i}}{R_{e}}$$

where *R_e_* is the resistance. The average current per unit area up to 8 h (*I_ave_*, mA/cm^2^) was calculated by dividing the mean current, obtained using the trapezoidal rule, by the effective diffusion area (2.01 cm^2^).

For cases 1–3, the *I_ave_* values during the experiments were 0.0498, 0.124, and 0.199 mA/cm^2^, as shown in Table 1.

### 2.8. Analytical Methods of LID·HCl

The concentration of LID·HCl in the collected samples was measured using HPLC. The Shimadzu HPLC system (Kyoto, Japan) was used in this study. It consists of a pump (LC-10AD_VP_), a UV detector (SPD-10A_VP_), a system controller (SCL-10A_VP_), a column oven (CTO-10A_VP_), an autosampler (SIL-10AF), and analysis software (LC solution).

For deproteinization, each sample solution was mixed with methanol at a 1:1 ratio, followed by centrifugation (15,000 rpm, 4 °C, 5 min; MX-301, Tomy Seiko Co. Ltd., Tokyo, Japan). The resulting supernatant (20 μL) was injected into the HPLC system for LID·HCl quantification.

Separation was performed using a TSKgel ODS-80Ts QA column (150 × 4.6 mm; Tosoh, Tokyo, Japan), maintained at 40 °C. The mobile phase consisted of 0.1% phosphoric acid containing 5.0 mM sodium *n*-dodecyl sulfate and acetonitrile (1:1). The flow rate was set at 1.0 mL/min. LID·HCl was detected at a UV wavelength of 262 nm. LID·HCl was quantified using a validated analytical method based on a calibration curve.

### 2.9. Statistics

Statistics for the skin permeation data were done by Tukey’s test.

## 3. Results

**Figure 5:**
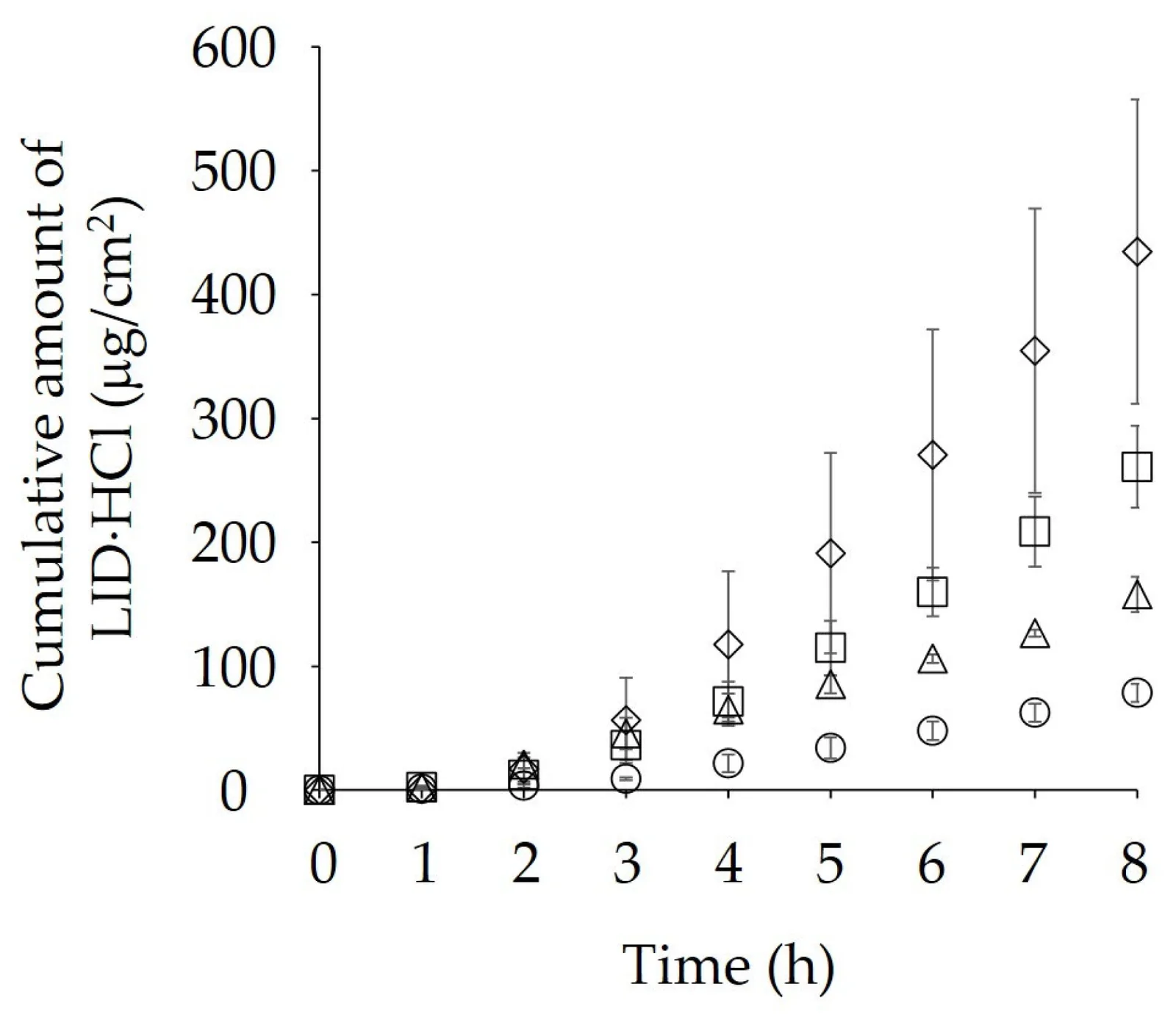
Time course of cumulative amount of LID·HCl permeated through skin under current application using a direct current generator (control and cases 1–3). Symbols are defined in Table 1. Each data point shows the mean ± S.D. (*n* = 3).

Figure 5 shows the skin permeation of LID·HCl (control and cases 1–3) when a constant current was applied using a direct current generator. All LID permeation amounts, including those described hereafter, are expressed as values calculated as LID·HCl. In this experiment, as shown in Table 1, a Ag electrode was used as the anode and an Ag/AgCl electrode as the cathode. When *I_ave_* values of 0.124, or 0.199 mA/cm^2^ were applied using the direct current generator, the skin permeation of LID·HCl was higher than that of the control (*p* < 0.05) (Figure 5). Although no significant difference was observed compared with the control, 0.0498 mA/cm^2^ showed a trend toward higher values than the control. Furthermore, LID·HCl permeation increased with increasing current intensity.

**Figure 6:**
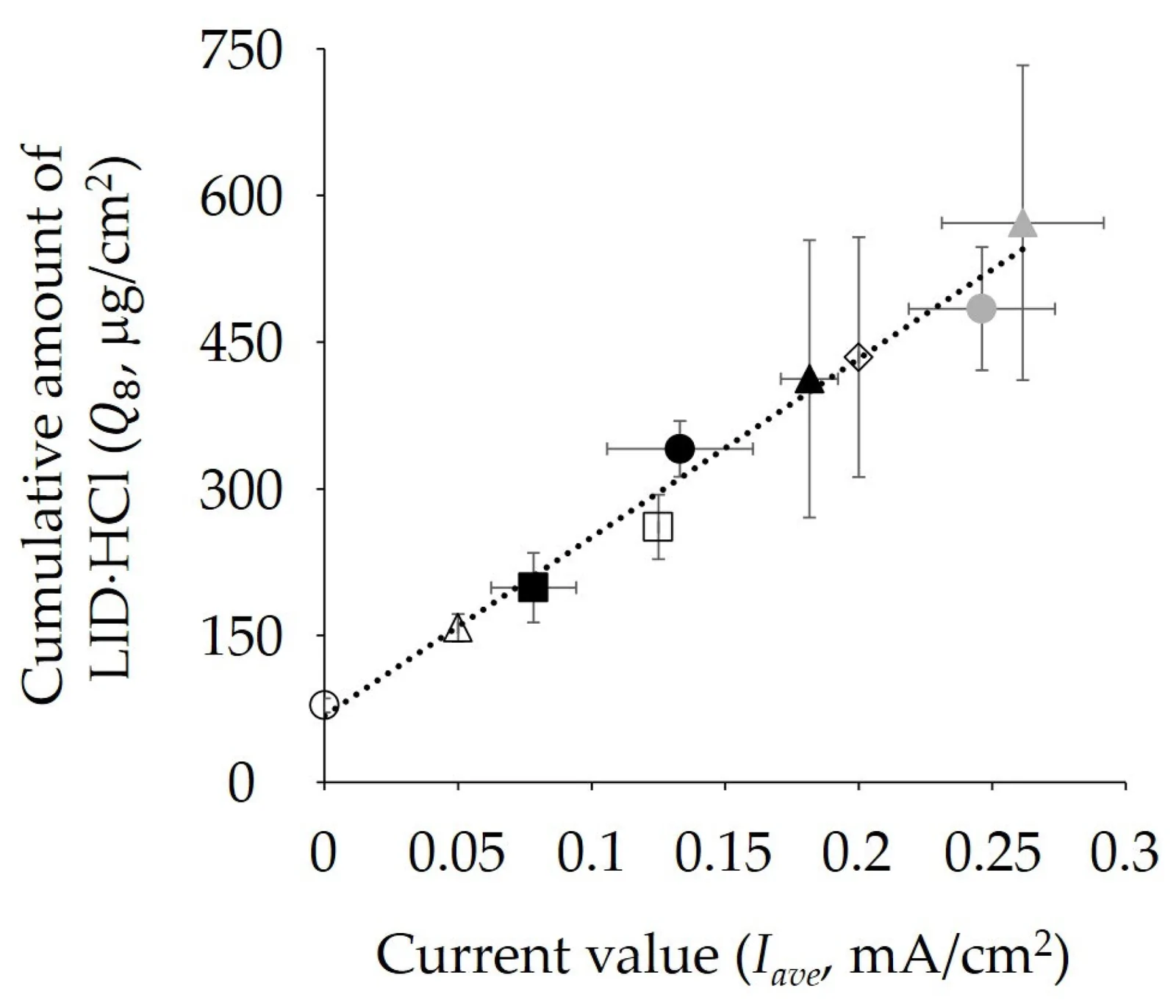
Relationship between the average current per unit area (*I_ave_*) and the 8 h cumulative skin permeation amount (*Q*_8_) for the control and cases 1–8. Symbols are defined in Table 1. Each data point shows the mean ± S.D. (*n* = 3).

Figure 6 shows the relationship between *I_ave_* and the cumulative amount of LID·HCl permeated/cm^2^ at 8 h (*Q*_8_) for the control and cases 1–3, indicated by open circles. The correlation coefficient between *Q*_8_ and *I_ave_* was 0.993, demonstrating an extremely strong correlation. The obtained correlation equation is shown Equation (4).

(4) $$Q_{8}={1744.1\;I}_{ave}+69.6$$

**Figure 7:**
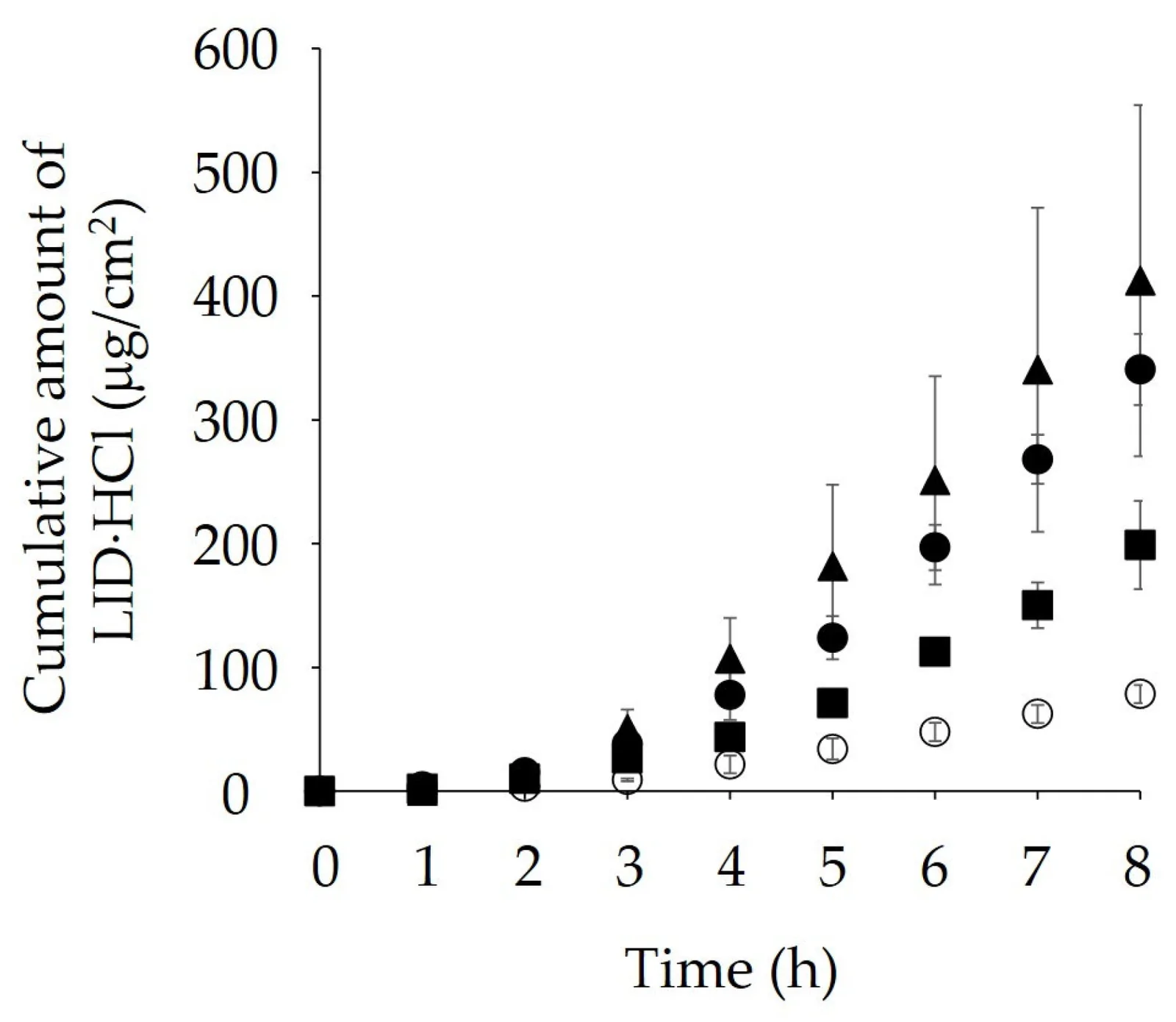
Time course of cumulative amount of LID·HCl permeated through skin under application of 1.5 V using a direct current generator or dry batteries (control and cases 4–6). Symbols are defined in Table 1. Each data point shows the mean ± S.D. (*n* = 3).

Figure 7 shows the 8 h cumulative amount of LID·HCl (*Q*_8_) under low-voltage conditions using a direct current generator and dry batteries. As shown in Table 1, in the control and cases 4–6, a Ag electrode was used as the anode and an Ag/AgCl electrode was used as the cathode. Although no significant difference was observed compared with the control, constant voltage of 1.5 V showed a trend toward higher values than the control. In addition, LID·HCl permeation was also increased in both the D battery and AA battery conditions compared with the control (*p* < 0.05). Interestingly, the LID·HCl permeation observed under AA battery conditions was higher than that obtained under the 1.5 V constant voltage condition using the direct current generator, despite the same nominal voltage being applied (*p* < 0.05). No significant difference in LID·HCl skin permeation was observed between the D battery and AA battery conditions.

**Figure 8:**
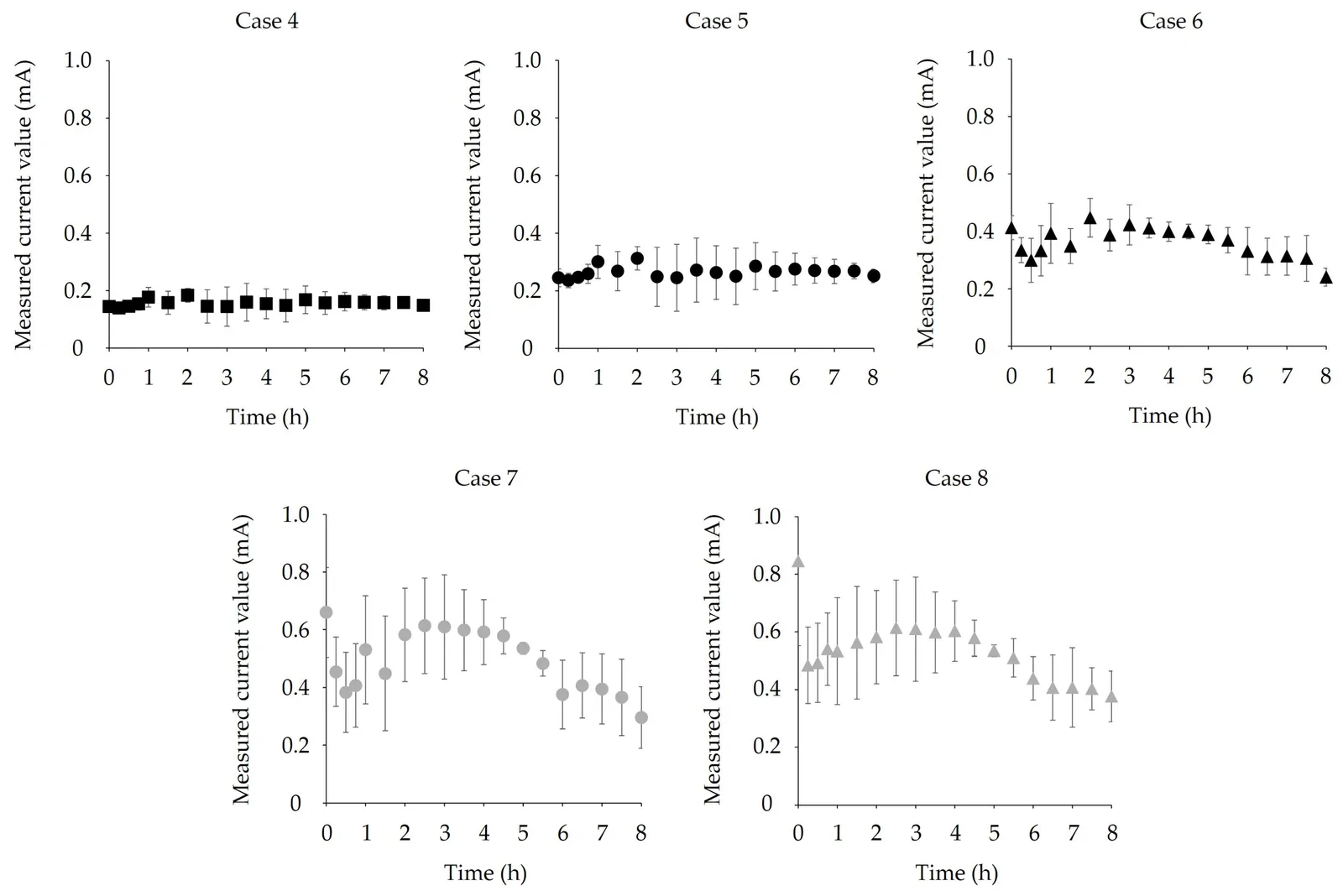
Measured current values recorded over 8 h using an oscilloscope (cases 4–8). Each data point shows the mean ± S.D. (*n* = 3).

Figure 8 shows the current values (*I_i_*) for cases 4–8, measured over 8 h using an oscilloscope. In cases 4–6, *I_i_* remained approximately constant throughout the entire measurement period from the start up to 8 h. These results indicate that, under the present experimental conditions, a stable current was maintained, suggesting that the electrical driving force did not change over time. Furthermore, to evaluate changes in battery voltage over time, a resistor (6.8 kΩ), which is comparable to the electrical resistance of the skin, was connected to AA and D batteries. The battery voltages measured immediately after the start of the experiment and after 8 h were 1.612 ± 0 V (mean ± S.D., *n* = 3) and 1.611 ± 0 V (mean ± S.D., *n* = 3) for the AA battery, and 1.606 ± 0.003 V (mean ± S.D., *n* = 3) and 1.614 ± 0.003 V (mean ± S.D., *n* = 3) for the D battery, respectively. No appreciable decrease in battery voltage was observed over the 8 h period for either the AA or the D battery. The voltage was measured using a digital multimeter (DT4253, Hioki E.E. Corporation, Nagano, Japan).

**Figure 9:**
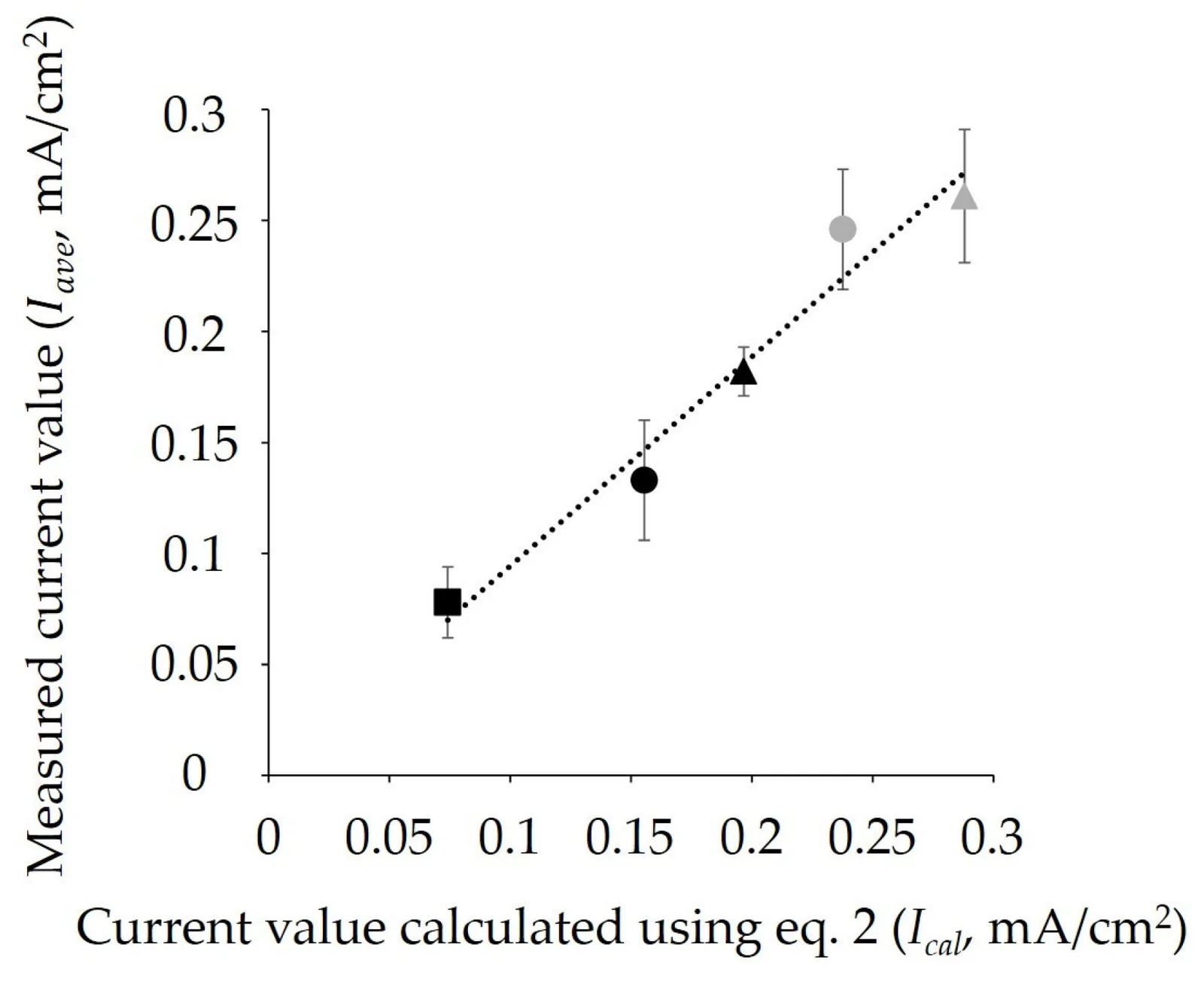
Relationship between the average current (*I_ave_*) and the calculated current (*I_cal_*) using Equation (4) (cases 4–8). Symbols are defined in Table 1. Each data point shows the mean ± S.D. (*n* = 3).

The experimentally determined *Q*_8_ values shown in Figure 7 were substituted into Equation (4) to calculate the average current per unit area, defined as *I_cal_*, and this was compared with the experimentally measured average current per unit area, *I_ave_*. The results are shown in Figure 9 (closed black symbols). As is evident from the figure, an approximately 1:1 relationship was observed between the measured average current (*I_ave_*) and the calculated current (*I_cal_*) for cases 4–6 (Figure 9, closed black symbols).

In addition, the relationship between *I_ave_* and *Q*_8_ under constant voltage conditions, using a direct current generator and dry batteries, is also shown by the closed black symbols in Figure 6. A strong correlation was observed between these variables (correlation coefficient = 0.988 for control and cases 1–6).

**Figure 10:**
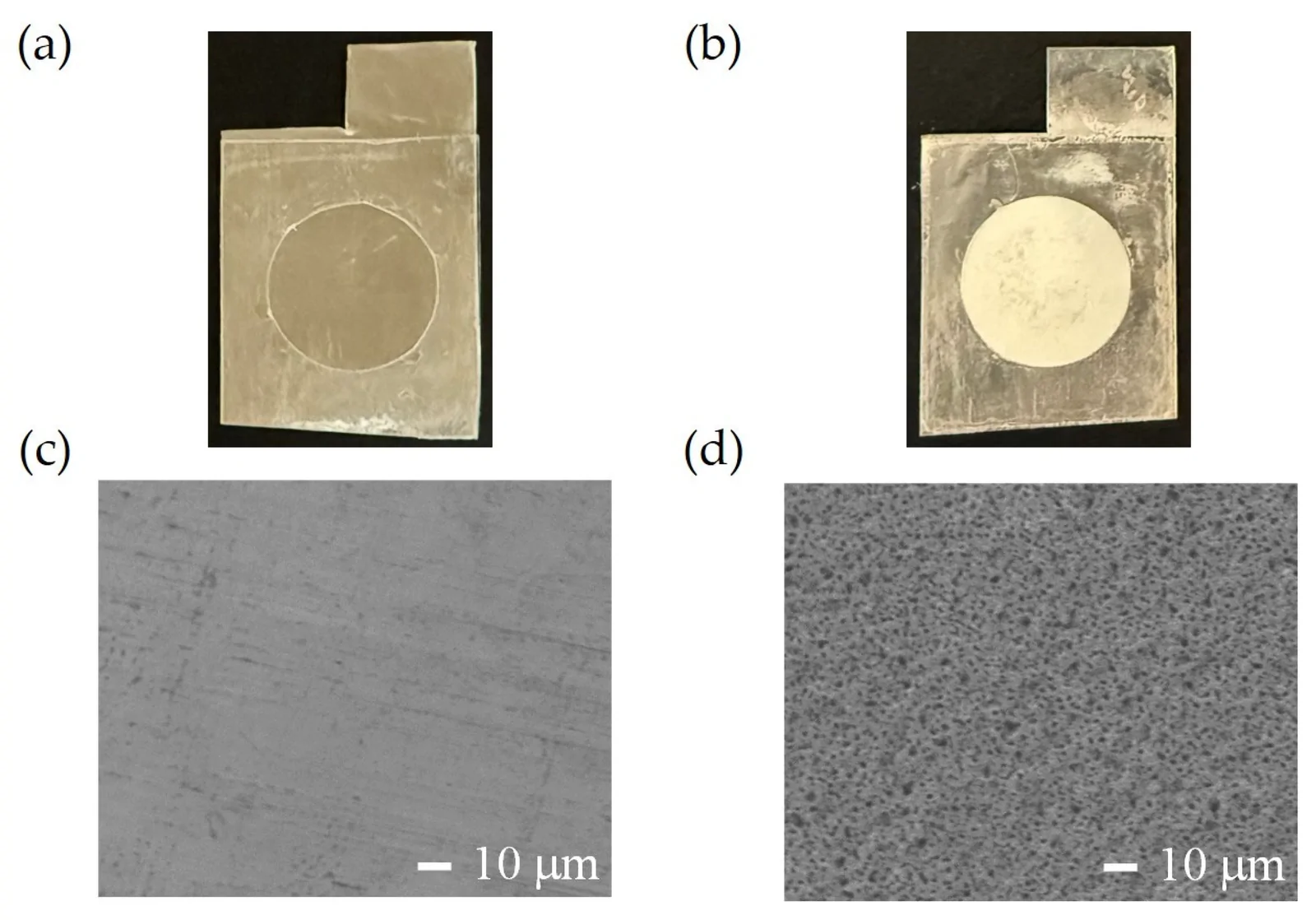
Photographs and SEM images of Ag and Ag* electrodes. (**a**) photograph of the Ag electrode, (**b**) photograph of the Ag* electrode, (**c**) SEM image of the Ag electrode (×400), and (**d**) SEM image of the Ag* electrode (×400).

Figure 10 shows photographs and SEM images of the Ag and Ag* electrodes. The surface of the Ag electrode was smooth (Figure 10a,c). On the other hand, the surface of the Ag* electrode, obtained by oxidizing the Ag electrode to form an Ag/AgCl electrode followed by reduction, exhibited irregular roughness, as observed in the SEM image at ×400 magnification (Figure 10d).

It should be noted that the SEM images in Figure 10c,d were enlarged and cropped on a personal computer to enhance the visibility of surface features. The original SEM images are provided in the Supplementary Materials. The maximum magnification available for SEM observation in this study was 400× because images obtained at higher magnifications could not be properly focused. Therefore, detailed evaluation of the surface pore structure was limited.

**Figure 11:**
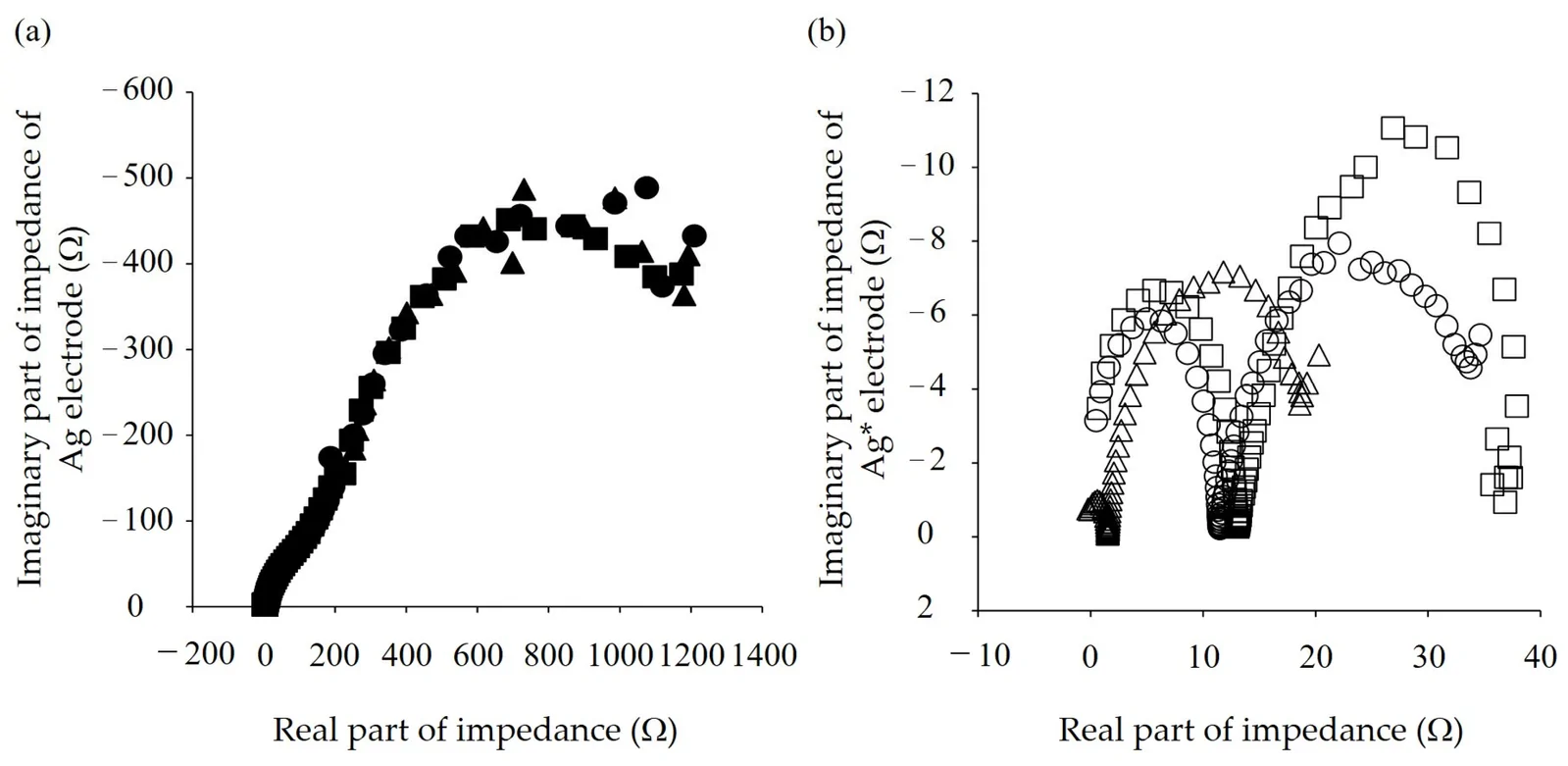
Representative Nyquist plot of the impedance spectra obtained by electrochemical impedance spectroscopy (EIS) for the Ag (**a**) and Ag* (**b**) electrodes. Each symbol represents individual electrode.

Figure 11 shows the Nyquist plots obtained from EIS measurements of the Ag and Ag* electrodes (working electrodes, WE). As shown in Figure 11a, the Ag electrode exhibited a deviation from the ideal semicircular behavior in the low-frequency region, suggesting a contribution from a diffusion process. Because the two-time-constant equivalent circuit could not adequately reproduce the impedance behavior in the low-frequency region, fitting was performed using an equivalent circuit model incorporating a Warburg element to account for the contribution of the diffusion process. In contrast, as shown in Figure 11b, although some variation was observed in the measured data for the Ag* electrode, arcs were observed in both the high- and low-frequency regions, suggesting an interfacial response with two-time constants. Therefore, the data were fitted using a two-time-constant equivalent circuit, *R_s_* − (*R*_1_||*C*_1_) − (*R*_2_||*C*_2_). For both equivalent circuit models, *R*_1_ was assigned to the resistance component arising from the surface layer formed on the electrode surface, such as an oxide film or adsorbed layer, whereas *R*_2_ was assigned to the resistance component associated with the charge-transfer reaction at the electrode/electrolyte interface. In this study, *R*_2_ was evaluated as the interfacial charge-transfer resistance. The *R*_1_ values were comparable between the Ag and Ag* electrodes, at 8.2 ± 3.2 Ω and 9.0 ± 6.4 Ω, respectively (mean ± S.D., *n* = 3). In contrast, the *R*_2_ value decreased from 44.1 ± 1.4 Ω for the Ag electrode to 19.3 ± 3.9 Ω for the Ag* electrode, corresponding to less than half of the value for the Ag electrode (*p* < 0.05).

**Figure 12:**
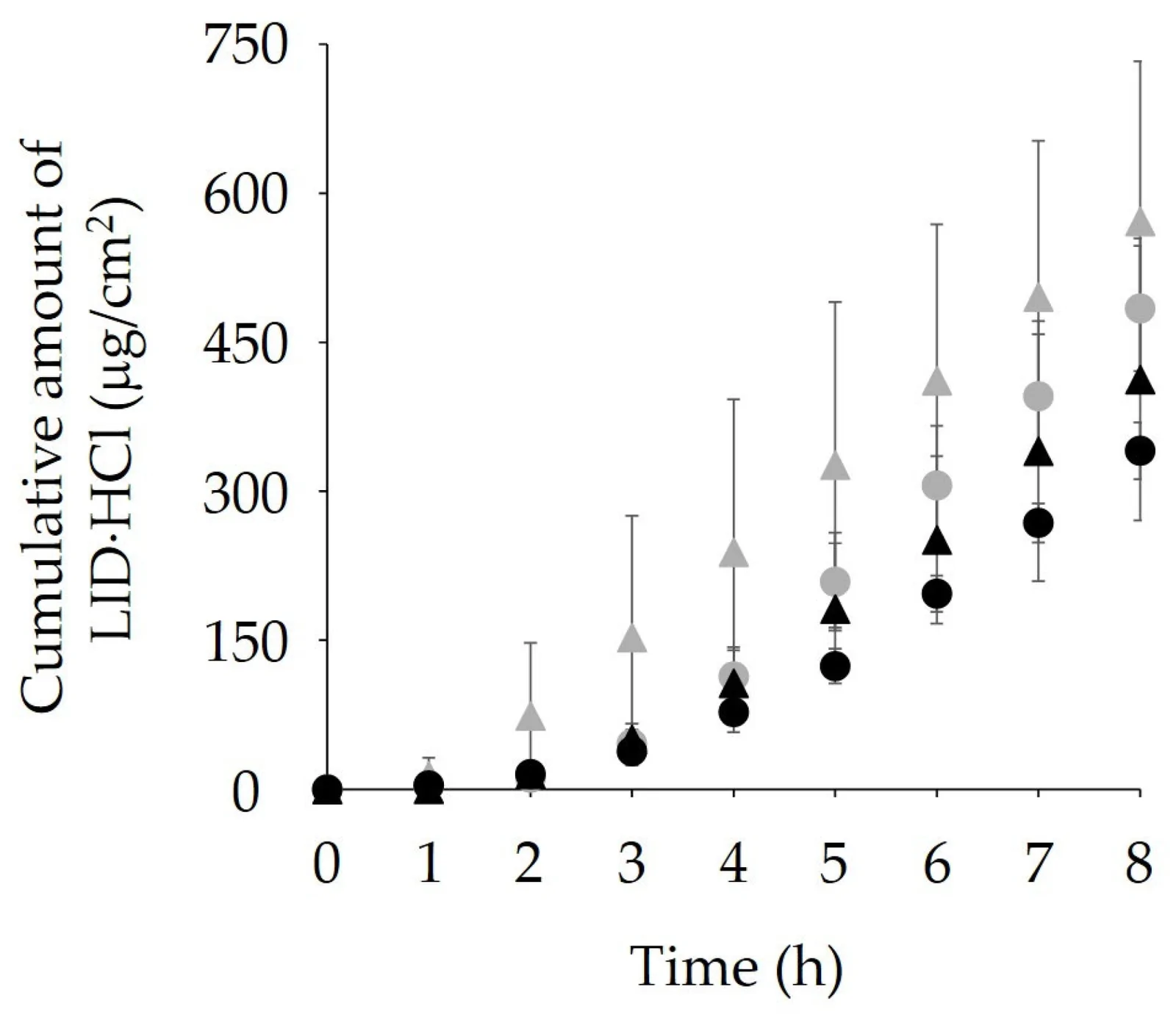
Time course of cumulative amount of LID·HCl permeated through skin when a D battery or AA battery was connected to a Ag or Ag* electrode and a Ag/AgCl electrode (cases 5–8). Symbols are defined in Table 1. Each data point shows the mean ± S.D. (*n* = 3).

Figure 12 shows the skin permeation of LID·HCl (cases 5–8) when a D battery or AA battery was connected to either the Ag or Ag* electrode in combination with an Ag/AgCl electrode. The cumulative 8 h permeation amounts (*Q*_8_) of LID·HCl under D battery and AA battery conditions were 484.1 ± 63.0 and 571.9 ± 161.1 μg/cm^2^, respectively (mean ± S.D., *n* = 3). Although no significant difference in LID·HCl permeation was observed between the Ag and Ag* electrode, the Ag* electrode tended to show higher LID·HCl permeation than the Ag electrode.

A strong correlation was observed between the average current (*I_ave_*) and *Q*_8_ for the Ag* and Ag/AgCl electrode system under D and AA battery conditions (cases 7 and 8; closed gray symbols in Figure 6), with an overall correlation coefficient of 0.990 for these cases.

For cases 4–8, the average current (*I_ave_*) was compared with the current calculated from the 8 h cumulative skin permeation based on Equation (4) (*I_cal_*). The resulting regression line was expressed as *I_ave_* = 0.925 *I_cal_*, with a slope close to unity (Figure 9, closed black and gray symbols). In addition, a high correlation coefficient of 0.982 was obtained between *I_ave_* and *I_cal_* for cases 4–8.

## 4. Discussion

In this study, we first investigated the effects of current conditions and electrode characteristics on the skin permeation of LID·HCl. The results showed that, under constant current conditions, the amount of permeation increased with increasing current intensity, and a very strong positive correlation (correlation coefficient = 0.993) was observed between *I_ave_* and *Q*_8_. These findings indicate that the permeation mechanism in iontophoresis is electrorepulsion, in which the amount of drug transported is proportional to the current density. In other words, positively charged LID^+^ permeates through the skin under the influence of an electric field, and the amount of permeation depends on the quantity of charge supplied. This relationship is consistent with Faraday’s law and also agrees with previous reports indicating that drug *flux* during iontophoresis is proportional to current density [44,45]. Furthermore, although this study employed animal skin, it is well known that there is little interspecies variation in the enhancement effect of transdermal permeation by iontophoresis [46]. From these results, it is suggested that the data obtained using porcine skin in this study may also be applicable to human skin. In addition, under both constant voltage conditions (1.5 V) and electromotive force conditions using batteries (AA battery), the transdermal permeation of LID·HCl increased compared with the control. Notably, the use of batteries resulted in higher permeation than that observed under constant voltage conditions. The lower permeation of LID·HCl observed with the constant-voltage generator compared with the battery-powered system may be related to the stable battery voltage throughout the 8 h experiment (AA battery: 1.612 ± 0 V at the start and 1.611 ± 0 V after 8 h; D battery: 1.606 ± 0.003 V at the start and 1.614 ± 0.003 V after 8 h; mean ± S.D., *n* = 3), as well as possible effects of the internal resistance and voltage-control mechanism of the constant-voltage generator. Under constant voltage conditions, the current is strongly influenced by the electrical resistance of the skin and its time-dependent changes, and thus is not maintained at a constant level, generally decreasing over time. In contrast, when batteries are used, a relatively higher current is maintained due to the balance between internal resistance and electrode reactions, resulting in greater drug permeation. In this study, the current calculated from the cumulative amount using the correlation equation (Equation (4)) was in good agreement with the average current measured directly using an oscilloscope, suggesting that the cumulative amount can be quantitatively predicted from the current value. Moreover, regardless of differences in constant current conditions, constant voltage conditions, battery types, or electrode structures, all data points were plotted on a single straight line representing the relationship between electric current and cumulative permeation.

In iontophoresis, the total amount of ions transported across the skin is proportional to the applied current (*I*), and the skin permeation *flux* of the target drug (*J_d_*), which takes into account its transport number (*t_d_*), can be expressed by the following Equation (5) [47]. Here, *Z_d_* is the ionic valence, and *F* is the Faraday constant.

(5) $$J_{d}=\frac{t_{d}I}{Z_{d}F}$$

Since *Z_d_* and *F* are constants, *J_d_* depends on *t_d_* and *I*. In the present study, *t_d_* represents the fraction of LID^+^ relative to the total amount of ions transported across the skin. If the current differs between the Ag and Ag* electrodes under constant-voltage conditions, the amount of Ag^+^ released from the silver electrode also differs, resulting in a change in *t_d_*. However, because this difference is considered to be small, we considered *I* to be the major factor influencing *J_d_* of LID·HCl. These results indicate that, regardless of differences in power supply conditions, the total amount of current passing through the system ultimately governs drug permeation.

In addition, the Ag/AgCl electrode system is widely used in iontophoresis because it enables stable current supply through reversible redox reactions and exhibits excellent biocompatibility [48,49]. The results of this study suggest that both the stability of electrode redox reactions and surface properties are critical factors.

The low-resistance electrode developed in this study can also be achieved by creating a rough electrode surface. The EIS results demonstrated that the use of the Ag* electrode significantly reduced the interfacial resistance. This may be attributed to an increase in the effective surface area of the electrode caused by the surface treatment, which facilitated electron-transfer reactions with the ferricyanide/ferrocyanide redox couple in the electrolyte. Since *R*_1_ showed little difference between the Ag and Ag* electrodes, the resistance component associated with the surface layer, such as an oxide film or adsorbed layer, was minimally affected by the surface treatment. In contrast, *R*_2_ decreased to approximately 44% of the value for the Ag electrode, indicating that the surface treatment markedly facilitated the charge-transfer reaction at the electrode/electrolyte interface. Therefore, the Ag* electrode was considered to reduce the interfacial charge-transfer resistance and enhance the electrochemical activity of the electrode.

In addition, a higher amount of charge passed during iontophoresis was observed for the Ag* electrode than for the Ag electrode. As described above, the Ag* electrode exhibited a lower interfacial charge-transfer resistance and enhanced electrochemical activity. Therefore, the oxidation reaction of Ag to Ag^+^ at the silver anode was considered to proceed more readily. Furthermore, the increase in the effective surface area and the number of active reaction sites resulting from the surface treatment may have contributed to more efficient electron-transfer reactions at the electrode/electrolyte interface, thereby allowing a greater amount of charge to be supplied to the system. Consequently, the increased amount of electric charges passed may have enhanced the transport of ions serving as charge carriers, potentially contributing to the increased drug permeation during iontophoresis. Taken together, these findings suggest that the increase in drug permeation resulting from the surface treatment was attributable, at least in part, to an increase in the effective electrode surface area and enhanced electrochemical activity resulting from the reduction in interfacial charge-transfer resistance. Further design and development focusing on current control using low-resistance electrodes are therefore considered particularly important for achieving stable and reproducible drug delivery in transdermal drug delivery systems employing iontophoresis. A limitation of this study is that the transdermal experiments were conducted for only 8 h. Therefore, the long-term working stability of the low-resistance electrode under prolonged application was not evaluated. Further studies are required to investigate the electrode performance over longer application periods.

On the other hand, iontophoresis is known to carry a risk of burns caused by electrical current, particularly under conditions of high current density or prolonged application [50]. Therefore, the acceptable current density in iontophoresis has been reported to be 0.5 mA/cm^2^ [51,52,53]. Although the current density applied in this study was below the reported safety threshold for electrical burns, the safety of prolonged continuous application was not evaluated. Therefore, skin safety and irritation associated with long-term use should be investigated in future studies.

## 5. Conclusions

This study demonstrated that the skin permeation of LID·HCl strongly depends on current density and that the permeation amount increases linearly with the current. It was also shown that the effects of constant voltage conditions, battery-driven systems, and differences in electrode surface structure are ultimately integrated into the current flowing through the skin. Furthermore, electrodes with a controlled surface structure improved current delivery efficiency and contributed to enhanced drug permeation. These results indicate that, in transdermal drug delivery using iontophoresis, both current control and electrode design are critical factors, and that highly efficient drug delivery can be achieved even with simple battery-powered systems. In the present study, we focused on the anodic electrode, particularly Ag, and demonstrated that a simple iontophoresis system powered by a battery could achieve high transdermal delivery of the cationic drug LID·HCl. However, it remains necessary to investigate whether similar improvements can be obtained with other cationic drugs. In addition, it is unclear whether the use of Ag would similarly increase the current and enhance the transdermal delivery of anionic drugs, or whether optimization of the cathodic electrode would be required to maximize electrorepulsion for anionic compounds. Therefore, further studies using a variety of drugs with different ionic properties are warranted. Furthermore, Ag* is currently prepared through two electrolysis steps, making the fabrication process laborious and unsuitable for large-scale production. Thus, the development of a simpler and more efficient preparation method, such as one based on chemical reactions, will also be necessary.

The effectiveness of the newly developed low-resistance electrode suggests that it may serve as an important design guideline for the practical application of iontophoretic transdermal drug delivery systems, including wearable devices and low-cost systems for pharmaceutical and cosmetic use.

## Figures and Tables

**Table 1:** Energizing devices, anode electrodes, and energizing conditions in the iontophoresis experiments.

Case No.	Energizing Device	Anode Electrode *^1^	Energizing Condition	Symbols in Section 3, Figures 5–9 and 12 *^4^
Control	—	—	—	○
1	Current generator	Ag	0.0498 mA/cm^2^ *^2^	△
2	Current generator	Ag	0.124 mA/cm^2^ *^2^	□
3	Current generator	Ag	0.199 mA/cm^2^ *^2^	◇
4	DC voltage generator	Ag	1.5 V *^3^	■
5	D battery	Ag	1.5 V *^3^	●
6	AA battery	Ag	1.5 V *^3^	▲
7	D battery	Ag*	1.5 V *^3^	●
8	AA battery	Ag*	1.5 V *^3^	▲

*^1^ Ag/AgCl electrode was used for the cathode in all iontophoresis experiments. *^2^ Constant current, and *^3^ Constant voltage (The rated voltage was specified to 1.5 V by the manufacturer). *^4^ The symbols represent the experimental conditions corresponding to each case in Figures 5–9 and 12: Control, ○; Case 1, △; Case 2, □; Case 3, ◇; Case 4, ■; Case 5, ●; Case 6, ▲; Case 7, ●; and Case 8, ▲.

## Data Availability

Dataset available on request from the authors.

## Supplementary Materials

The following supporting information can be downloaded at https://www.mdpi.com/article/10.3390/molecules31183269/s1, Figure S1: SEM images of Ag and Ag* electrodes. (a) SEM image of the Ag electrode (×400), and (b) SEM image of the Ag* electrode (×400).

## Abbreviations

The following abbreviations are used in this manuscript:

LID·HCl	Lidocaine Hydrochloride
SEM	Scanning Electron Microscope
*V_i_*	Voltage value at each time point
*R_e_*	Resistance value
*I_i_*	Current value at each time point
*I_ave_*	Average current per unit area up to 8 h
*I_cal_*	Calculated current using Equation (4)
*Q*_8_	Cumulative amount at 8 h
