# Supplementary material for: Usefulness of Low-Resistance Electrodes Used in Iontophoresis to Enhance Skin Permeability of Drugs
Source: Molecules. 2026 Sep 15;31(18):3269. doi: 10.3390/molecules31183269 (PMC13609247; doi:10.3390/molecules31183269)
Supplement: Supplementary file 1 [file molecules-31-03269-s001.zip › Figure S1.pdf]

(a) (b)

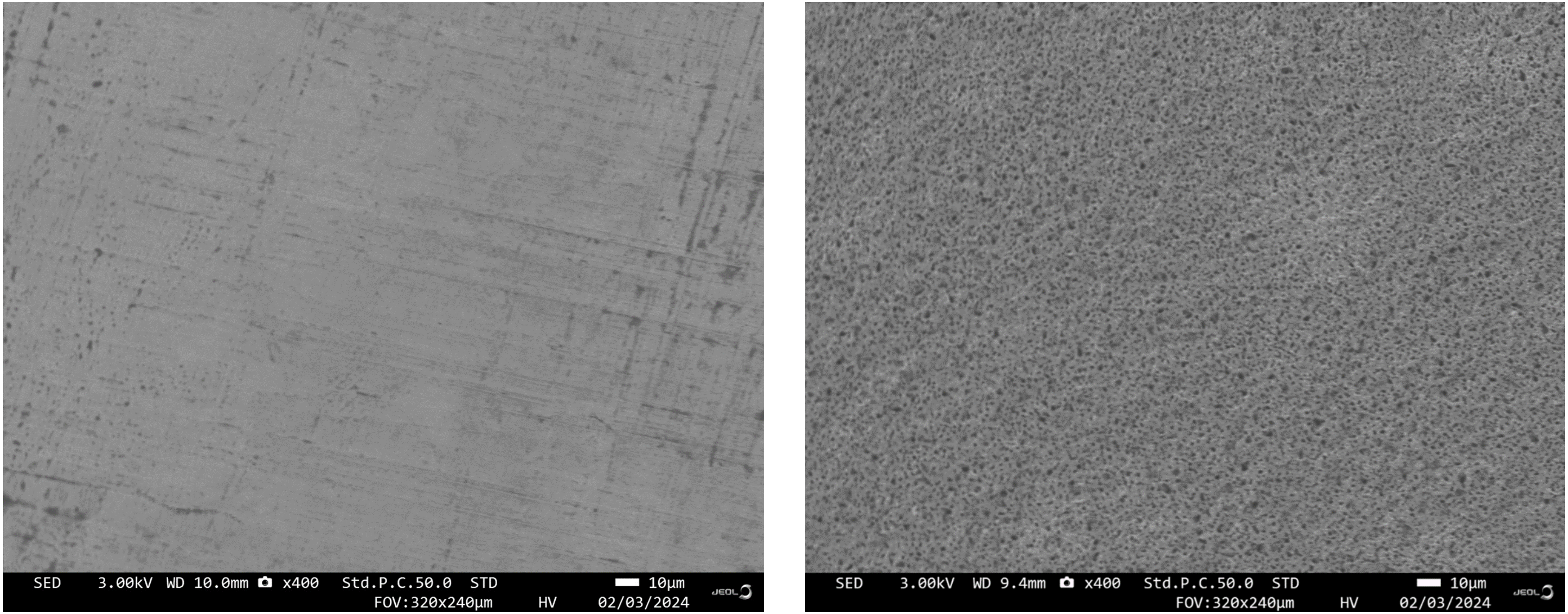

SEM images of Ag and Ag* electrodes. (a) SEM image of the Ag electrode (×400), and (b) SEM image of the Ag* electrode (×400).
